# Antidiabetic and Hypolipidemic Activities of *Curculigo latifolia* Fruit:Root Extract in High Fat Fed Diet and Low Dose STZ Induced Diabetic Rats

**DOI:** 10.1155/2013/601838

**Published:** 2013-05-15

**Authors:** Nur Akmal Ishak, Maznah Ismail, Muhajir Hamid, Zalinah Ahmad, Siti Aisyah Abd Ghafar

**Affiliations:** ^1^Nutricosmeceutical and Nutrigenomic Programme, Laboratory of Molecular Biomedicine, Institute of Bioscience, Universiti Putra Malaysia, 43400 UPM Serdang, Selangor Darul Ehsan, Malaysia; ^2^Department of Nutrition and Dietetics, Faculty of Medicine and Health Sciences, Universiti Putra Malaysia, 43400 UPM Serdang, Selangor Darul Ehsan, Malaysia; ^3^Department of Microbiology, Faculty of Biotechnology and Biomolecular Sciences, Universiti Putra Malaysia, 43400 UPM Serdang, Selangor Darul Ehsan, Malaysia; ^4^Chemical Pathology Unit, Department of Pathology, Faculty of Medicine and Health Sciences, Universiti Putra Malaysia, 43400 UPM Serdang, Selangor Darul Ehsan, Malaysia

## Abstract

*Curculigo latifolia* fruit is used as alternative sweetener while root is used as alternative treatment for diuretic and urinary problems. The antidiabetic and hypolipidemic activities of *C. latifolia* fruit:root aqueous extract in high fat diet (HFD) and 40 mg streptozotocin (STZ) induced diabetic rats through expression of genes involved in glucose and lipid metabolisms were investigated. Diabetic rats were treated with *C. latifolia* fruit:root extract for 4 weeks. Plasma glucose, insulin, adiponectin, lipid profiles, alanine aminotransferase (ALT), gamma glutamyltransferase (GGT), urea, and creatinine levels were measured before and after treatments. Regulations of selected genes involved in glucose and lipid metabolisms were determined. Results showed the significant (*P* < 0.05) increase in body weight, high density lipoprotein (HDL), insulin, and adiponectin levels and decreased glucose, total cholesterol (TC), triglycerides (TG), low density lipoprotein (LDL), urea, creatinine, ALT, and GGT levels in diabetic rats after 4 weeks treatment. Furthermore, *C. latifolia* fruit:root extract significantly increased the expression of *IRS-1*, *IGF-1*, *GLUT4*, *PPAR**α***, *PPAR**γ***, *AdipoR1*, *AdipoR2*, *leptin*, *LPL*, and *lipase* genes in adipose and muscle tissues in diabetic rats. These results suggest that *C. latifolia* fruit:root extract exerts antidiabetic and hypolipidemic effects through altering regulation genes in glucose and lipid metabolisms in diabetic rats.

## 1. Introduction

Type 2 diabetes mellitus is a metabolic disorder which causes hyperglycemia [1] due to defect in insulin secretion and insulin resistance [2]. It results from failure of pancreatic *β*-cell to secrete insulin sufficiently in response to elevate blood glucose [3]. In early stage of type 2 diabetes mellitus, peripheral tissues such as liver, muscle, and adipose tissues are not sensitive towards insulin [4] and they cause common symptoms such as increased thirsty, frequent urinary, ketonuria and ketonemia in diabetic patients [5]. In a long-term of insulin resistance period, it will lead to chronic hyperglycemia and hyperlipidemia in diabetic patients [6]. Therefore, if no prevention action is taken, diabetes can lead to several complications such as heart attack, nephropathy, retinopathy, and neuropathy [7].

Till now, numerous types of diabetic models are used for screening antidiabetic properties of plants. These diabetic models are developed through several methods either through genetic or chemically induced diabete [8]. There are genetic models of diabetes known as db/db mouse and Zucker diabetic fatty (ZDF) rats which develop similar features as in human type 2 diabetes [9]. However, the development of diabetes in these rats is due to genetic and this is unlikely in humans. Besides, these rats are expensive to be used as diabetic models for pharmacological screening [10]. Meanwhile, development of diabetic rats following streptozotocin (STZ) injection also presents hyperglycemia similar to human type 2 diabetes mellitus [11]. However, this method only develops insulin deficiency rather than insulin resistance in the model [12]. Despite that, the pattern of disease progress did not appear to be similar to diabetic situation in human type 2 diabetes mellitus. Thus, several researchers have been investigating to find a better diabetic model for type 2 diabetes mellitus by modifying the existing method. Recently, many studies reported that rats induced with high fat diet with combination of (STZ) have developed similar situation as type 2 diabetes progress in humans. Diets containing high fat will cause insulin resistance in peripheral tissues due to lipotoxicity [13]. Meanwhile, low dose of STZ has known to induce mild defect in insulin secretion, which is similar to the characteristics of the later stage of type 2 diabetes [14]. Combination of high fat diet with low dose STZ has successfully mimicked natural progress of diabetes development as well as metabolic features in human type 2 diabetes [15, 16]. Apart from that, these models are also cheaper, easy to develop, and practical for pharmacological screening [17].

Currently, there are five major classes of therapeutic drugs that have been used to treat diabetes through several target sides: sulfonylureas, biguanides, thiazolidinediones, meglitinide, and *α*-glucosidase inhibitor. However, according to Bastaki (2005), combination of two drugs such as metformin and sulfonylurea can increase the hypoglycemic activity where it treats diabetes through two different modes of action [18]. In spite of antidiabetic drugs effectiveness, prolonged usage of it will cause adverse effect. Sulfonylureas have been reported to cause hypoglycemia, increase in body weight, gastrointestinal (GI) disturbance, and headache to the user [19]. Besides, metformin could cause abdominal pain, diarrhea, nausea, and lactic acidosis to the diabetic patient [20]. In other diabetic drug that causes adverse side effects is thiazolidinediones as it causes hepatotoxicity after long-time usage [21]. Apart from adverse side effect, antidiabetic drug also has limited mode of action. Available diabetic drugs only show single mode of action in treating diabetes and then need to combine with another class of antidiabetic drug to make these drugs more efficient such as combination of metformin and sulfonylurea [18].

Although there was a plenty amount of antidiabetic drugs available, the numbers of type 2 diabetes mellitus patients are still increased constantly. The incidence of T2DM has become worldwide epidemic and there are approximately 246 million people who are suffering from this disease [22]. The highest rate of diabetic population is in India with current figure 40.9 million and followed by China with 39.8 million. Furthermore, Pakistan, Japan, USA, Russia, and Germany are also widely affected with diabetes [22]. This number is continually increasing and it might be due to antidiabetic drug adverse side effect and also due to drug limitation action. 

There is a dramatic revival of interest in using natural sources in treating diabetes due to side effects of prolonged consumption of therapeutic drugs. Asian countries such as India and China are already known for their contributions toward the usage of plant medicine in preventing and overcoming diabetes problems [23, 24]. More than 4000 plants have been studied and identified to have hypoglycemic effect through several mechanisms of antidiabetic activity. Some plants acts through either secretagogues or insulin mimetic properties such as *Momordica charantia*, *Aloe vera,* and *Allium sativum* [25–27]. Studies have reported that these plants have the ability to reduce blood glucose and improve insulin secretion [28]. Meanwhile, according to Malviya et al. (2010), secondary metabolites from plants such as phenolic, alkaloids, and glycosides are the ones which are implicated as having antidiabetic effect [26]. Among those secondary metabolites, phenolic compounds are the ones which are abundantly present in plants and are demonstrated to have antioxidant, antidiabetic, and antiobesitiy properties [29].

*Curculigo latifolia* (Dryand, ex W. T.Aiton) is a shrub tree that mainly grows under rubber tree and it is also known as Lemba among local community in Malaysia [30]. This plant belongs to the Hypoxidaceae family. To date, there are about 20 species of *Curculigo* that have been identified and *C. latifolia* and *C. capitulate* are mostly distributed in Malaysia [31]. This shrub tree consists of berry-like fruit and this fruit exhibits both sweet tasting and taste modifying activities [32]. Curculin and neoculin have been identified as proteins that possess those activities [33]. Despite *C. latifolia* is sweet and can be used as alternative sweetener for diabetic patient, and there is no scientific study on *C. latifolia* as antidiabetic agent. Preliminary study that has been conducted in our laboratory showed that *C. latifolia* fruit and root extracts have the highest antioxidant activity where the IC_50_ for both of the fruit and root extracts is 1.0 mg/mL. In spite of high antioxidant, fruit and root also consist of high phenolic content, 95 mg GAE/100 g extract and 90 mg GAE/100 g extract. Both data revealed that there is positive correlation between total phenolic content and antioxidant activity in fruit and root. Besides, an *in vitro* study that has been conducted in our laboratory indicates that *C. latifolia* fruit and root extracts have antidiabetic activity by increased insulin and adiponectin secretion in cell lines. *C. latifolia* fruit and root extracts also significantly increased glucose uptake activity in 3T3 adipocytes and L6 myotubes cell lines (patent pending). The present study was performed to determine antidiabetic and hypolipidemic activities of *C. latifolia *fruit:root extract in HFD and low dose STZ induced diabetic rats by evaluating the potential of this plant to regulate expression of genes involved in glucose and lipid metabolisms.

## 2. Materials and Methods

### 2.1. Preparation of *C. Latifolia* Fruit:root Extract

*C. latifolia* plant was collected from Beranang, Selangor, Malaysia. It was identified by the taxonomist from the Biodiversity Unit in the Institute of Bioscience, Universiti Putra Malaysia with voucher number SK 1709/09. The fruits were plucked at the apex of *C. latifolia* stem, cleaned with tap water, blotted with tissue paper, and stored at −20°C until further use. Roots were cleaned with tap water and immediately dried for overnight in an air oven (Memmert, Schwabach, Germany) at 40°C. Dried roots were grounded to fine powder using electric grinder (Philips, Malaysia). Powdered root was sealed in plastic bags and kept at 4°C until further use. 

#### 2.1.1. Extraction of *C. Latifolia* Fruits : Root

Fifty grams of fresh *C. latifolia* fruits was mashed using mortar and pestle. Mashed fruits were extracted with 2000 mL of distilled water. Meanwhile, 50 g of *C. latifolia* root powder was soaked in 2000 mL of distilled water. Both extractions were extracted 24 h with continuous stirring at room temperature. This extract was filtered through Whatman number 1 filter paper and the filtrate was collected and lyophilized. The lyophilized sample was kept at −80°C until further use. In this study, fruit and root extracts were mixed at 1 : 1 ratio for rats treatment.

### 2.2. Preparation of High Fat Diet (HFD)

The normal pallet diet from Miba Mansura (Malaysia) consists of 46% of cornstarch, 26% of palm kernel meal, 4% of soybean oil, 3.5% of minerals mixture, 1% of vitamins mixture, 0.25% of choline bitartrate, and 0.18% of L-cystine. The nutrient composition is shown as Table 1. The HF diet was formulated based on the composition provided by Levin et al. (1989) [34]. It will be prepared from a mixture of 50% normal rat chow pellet, 24% of corn oil (Mazola brand), 20% of full-cream milk powder (NESPRAY brand from Nestlé), and 6% sugar.

### 2.3. Animal Study

 Forty-two male Sprague-Dawley rats weighing 160–180 g each were housed individually in polypropylene cages and maintained under controlled room temperature (22 ± 2°C) and humidity (55 ± 5%) with 12:12 h light-dark cycle. All experimental protocols for animal care and use were approved by the Animal Care and Use Committee (ACUC) of the Faculty of Medicine and Health Sciences, Universiti Putra Malaysia (project approval number UPM/FPSK/PADS/BR-UUH/0030). Upon receipt, rats were acclimatized for a week with free access of water and normal pellet diet. After acclimatized, normal rats (*n* = 6) were maintained on normal pellet diet while remaining rats were given high fat diet to induce obesity. This treatment was conducted for 4 weeks. After 4 weeks, obese rats were anesthetized with diethyl ether after being fasted overnight and injected with 40 mg/kg bw of STZ via intravenous to induce type 2 diabetes. Diabetic rats (fasting blood glucose level > 170 mg/dL after 7 days of STZ injected) were randomly divided into 5 groups (Groups 3, 4, 5, 6, and 7) and each group consists of 6 rats. Below are the lists of the rat groups for this study.

 Group 1: normal (normal pellet diet, untreated) rats.
 Group 2: obese (high fat-fed diet, obese, untreated) rats.
 Group 3: diabetic control (high fat-fed diet, induced with STZ, diabetic, untreated) rats.
 Group 4: diabetic test rats (high fat-fed diet, induced with STZ, diabetic) treated with 50 mg/kg bw of *C. latifolia* fruit:root extracts.
 Group 5: diabetic test rats (high fat-fed diet, induced with STZ, diabetic), treated with 100 mg/kg bw of *C. latifolia* fruit:root extracts.
 Group 6: diabetic test rats (high fat-fed diet, induced with STZ, diabetic), treated with 200 mg/kg bw of *C. latifolia* fruit:root extracts.
 Group 7: diabetes test rats (high fat-fed diet, induced with STZ, diabetic) treated with 10 mg/kg bw of glibenclamide.

Treatment on diabetic rats was done for 4 weeks. Body weight of each rat was recorded before (0 week) and after 4 weeks of treatment. At the end of the experimental period, all rats were fasted for 15 h prior to sacrifice. Blood samples were collected by cardiac puncture. Meanwhile, adipose and muscle tissues were excised and stored at −80°C prior used.

### 2.4. Biochemical Parameter Analysis of Blood

Blood samples were collected using K_2_EDTA blood collection tube (BD Diagnostics, Franklin Lakes, NJ, USA). Plasma was collected after blood was centrifuged at 3000 rpm for 10 minutes [35]. Biological assay such as glucose, total cholesterol (TC), triglycerides (TG), low density lipoprotein (LDL), high density lipoprotein (HDL), urea, creatinine, alanine aminotransferase (ALT), and *γ*-glutamyltransferase (GGT) was measured using Selectra XL clinical chemistry analyzer (Vital Scientific, the Netherlands). Insulin level was measured using rat insulin ELISA kit (Mercodia AB, Uppsala, Sweeden) with rat insulin as a standard. Adiponectin level was measured using BioVision rat adiponectin ELISA assay (BioVision Inc., Mountain View Milpitas, CA, USA).

### 2.5. Quantification of Different Expression Genes between Group Treatments Using GenomeLab

The white adipose tissues (retroperitoneal, subcutaneous, and epididymal) and muscle tissues were harvested from rats. Frozen tissues were thawed and homogenized and total RNA was extracted using RiboPure isolation of high quality total RNA (Ambion, USA) according to manufacturer's instructions. Reverse transcription and PCR procedures were performed according to GenomLab GeXP kit protocol (Beckman Coulter, USA) using XP Thermal Cycler (Bioer Technology, Germany). The amplicons from PCR reaction were used for quantification of different expression genes between group treatments using GenomeLab GeXP Start kit. Samples were prepared and were added to the appropriate wells of 96-well sample microplate. All the samples were run in triplicates. Besides, all the data were analyzed using Express analysis software where fragment data is easily identified. Multiplex genes were normalized with 18S by dividing the peak area of each gene by peak area of 18S gene. The expression level was calculated according to the following formula:

(1) Fold  change =Normalized  data  of  the  gene  from  treated  samplesNormalized  data  of  the  gene  from  untreated  samples.

Primer sequences for all rat genes were designed using eXpress designer module of the GenomeLab eXpress Profiler software based on gene sequences from GeneBank database (Table 2).

### 2.6. Statistical Analyses

All results are expressed as the mean ± standard deviation. The data were analyzed using one-way analysis of variance (ANOVA), followed by Tukey's post hoc test. Level of significance was set at *P* < 0.05. 

## 3. Results

### 3.1. Energy Contributed from HFD

In the present study, diabetic rats were developed using high fat diet. The high fat diet was formulated according to Levin et al.'s [34] method. The nutrient composition of NPD and HFD used in this experiment was mentioned in Table 1. Meanwhile, Table 3 shows the energy contributed from NPD and HFD. Energy contributed from fat sources in HFD diet was 56.9% and it indicates that there was 47.4% increase in fat composition in HFD compared to NPD. In spite of fat, energy contributed by protein and carbohydrate was 11.9% and 31.2%. 

### 3.2. Body Weight

**Figure 1:**
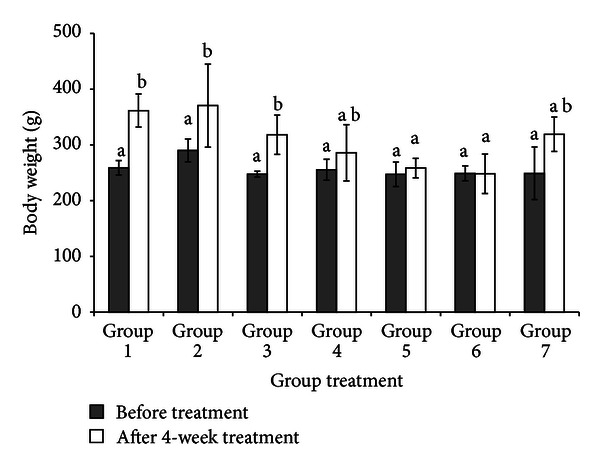
Rats body weight before (0 week) and after treatment (4 week). Body weight of normal rats (Group 1), obese rats (Group 2), diabetic control rats (Group 3), diabetic rats treated with 50 mg/kg bw of *C. latifolia* fruit:root (Group 4), diabetic rats treated with 100 mg/kg bw of *C. latifolia* fruit:root (Group 5), diabetic rats treated with 200 mg/kg of *C. latifolia* fruit:root (Group 6), and diabetic rats treated with 10 mg/kg bw of glibenclamide (Group 7). Columns represent the mean ± S.D. (*n* = 6).  ^a,b^significantly different at *P* < 0.05.

Body weight from week 0 to week 4 of normal, obese, untreated diabetic and treated diabetic rats increased significantly (*P* < 0.05). In normal rats (Group 1), body weight increased by 20% followed by 30% in obese rats (Group 2). Meanwhile, body weight of diabetic control rats (Group 3) increased by 6%. However, body weight of diabeticrats treated with *C. latifolia* fruit:root extract increased by 12% (Group 4), 9% (Group 5), and 7% (Group 6) after 4 weeks of treatment. Body weight of diabetic rats in group 7 which has been treated with glibenclamide increased by 19% (Figure 1).

### 3.3. Plasma Glucose Level

After 4 weeks of intervention, glucose level in normal group was maintained in normal range throughout the experiment although it increased slightly by 2.4%. Meanwhile, glucose level in obese (Group 2) and diabetic control (Group 3) rats was significantly (*P* < 0.05) increased by 18.6% and 15.2% by the end of the study. However, glucose level was decreased in treated diabetic rats. The most significant (*P* < 0.05) reduction was showed in diabetic rats treated with 200 mg/kg bw of *C. latifolia* fruit:root extracts (Group 6) followed by 100 (Group 5) and 50 (Group 4), 64.5% > 54.3% > 51.6%. Meanwhile, diabetic rats treated with 10 mg/kg bw of glibenclamide show 32.4% reduction in glucose level (Table 4).

### 3.4. Plasma Lipid Profiles

**Figure 2:**
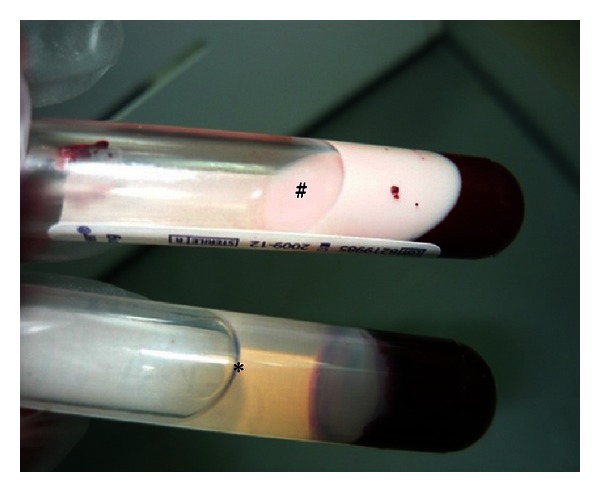
Picture of plasma lipid in diabetic control and normal rats. Diabetic control rats are marked with (#) and normal rats with (∗).

Lipid profiles in normal rats were in normal range. However, plasma TC, LDL, and TG levels in obese rats (Group 2) were significantly higher (*P* < 0.05) while HDL level was lower than that normal rats (Group 1) after 4 weeks of study (Table 5). Diabetic control rats (Group 3) also showed a similar pattern to obese rats where plasma TC, TG, and LDL levels were increased by 56.2%, 52.6%, and 75.4% compared to normal rats. However, plasma HDL level in diabetic control rats was decreased by 51%. The higher level of lipid in diabetic control rats can be seen in plasma. Plasma lipids in diabetic control rats were higher and plasma colour has turned to opaque colour instead of clear (Figure 2). The posttreatment levels of TC, TG, and LDL of treated groups were significantly decreased compared to pretreatment levels. Diabetic rats in Groups 4, 5, and 6 showed a significant (*P* < 0.05) reduction in plasma TC, TG, and LDL levels compared to diabetic rats treated with glibenclamide. Besides, HDL level also increased after 4 weeks of treatment in diabetic rats in Groups 4, 5, and 6.

Thus, hypocholesterolemia effect has been found the be higher in 200 mg then followed by 100 mg and 50 mg of *C. latifolia* fruit:root extracts. The most striking result emerging from this study is that lipid content in plasma was ameliorated when treated with *C. latifolia* extracts. As consequence, the opaque colour in plasma turned into clear in plasma sample in Groups 4, 5, and 6.

### 3.5. Plasma Insulin and Adiponectin

**Figure 3:**
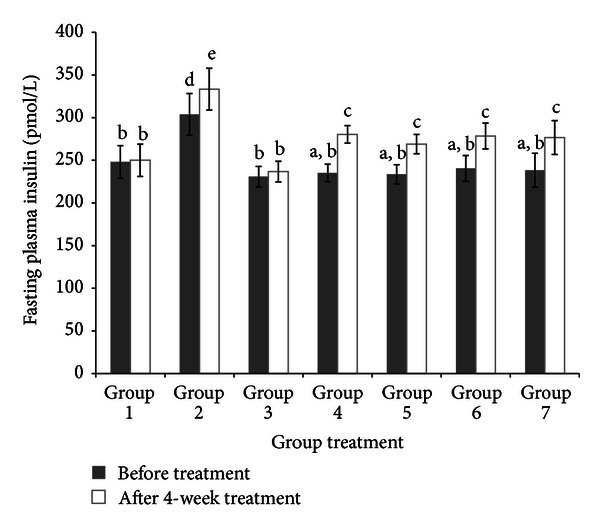
Fasting plasma insulin before (0 week) and after treatment (4 week). Insulin level of normal rats (Group 1), obese rats (Group 2), diabetic control rats (Group 3), diabetic rats treated with 50 mg/kg bw of* C. latifolia* fruit:root (Group 4), diabetic rats treated with 100 mg/kg bw of *C. latifolia* fruit :  root (Group 5), diabetic rats treated with 200 mg/kg of *C. latifolia* fruit:root (Group 6), and diabetic rats treated with 10 mg/kg bw of glibenclamide (Group 7). Columns represent the mean ± SD (*n* = 6).  ^a,b,c^significantly different at *P* < 0.05.

 The changes in insulin level after 4-week intervention are shown in Figure 3. There was no significant (*P* < 0.05) change in plasma insulin level in normal and diabetic control rats before and after 4 weeks of study. However, plasma insulin level in obese rats significantly increased. Four weeks of treatment with *C. latifolia* fruit:root extract and glibenclamide had increased insulin levels in diabetic rats. There was 16% of insulin increasing in rats in Group 4 followed by 13% in Group 5, 12% in Group 6, and 11% in Group 7.

**Figure 4:**
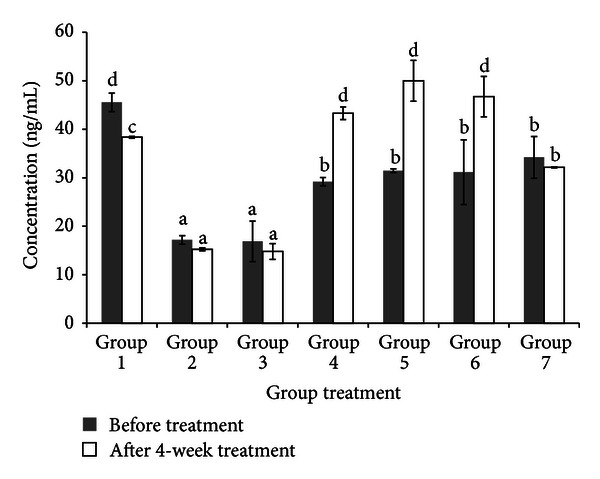
Fasting plasma adiponectin before (0 week) and after treatment (4 week). Adiponectin level of normal rats (Group 1), obese rats (Group 2), diabetic control rats (Group 3), diabetic rats treated with 50 mg/kg bw of *C. latifolia* fruit:root (Group 4), diabetic rats treated with 100 mg/kg bw of *C. latifolia* fruit:root (Group 5), diabetic rats treated with 200 mg/kg of *C. latifolia* fruit:root (Group 6), and diabetic rats treated with 10 mg/kg bw of glibenclamide (Group 7). Columns represent the mean ± SD. (*n* = 6).  ^a,b,c^significantly different at *P* < 0.05.

There are remarkable changes in adiponectin level in obese and diabetic control rats compared to normal rats (Figure 4). Adiponectin level in obese and diabetic control rats was lower than that normal rats. However, *C. latifolia* fruit:root extract prevents further decrease in adiponectin in diabetic rats. Adiponectin level was significantly (*P* < 0.05) increased in diabetic rats treated by *C. latifolia* fruit:root extract. Group 5 increased adiponectin level by 56% followed by Group 4 (48%) and Group 6 (41%). Besides, in Group 7 there is no significant difference in adiponectin level after 4 weeks of intervention.

### 3.6. Plasma ALT, GGT, Urea, and Creatinine

Results in Table 6 indicate that ALT, GGT, urea, and creatinine levels significantly (*P* < 0.05) increased in obese and diabetic control rats when compared to normal rats. However, there is no significant difference (*P* < 0.05) in ALT, GGT, urea, and creatinine level in diabetic control (Group 3) rats when compared to obese rats. Four weeks of intervention with *C. latifolia* fruit:root extract have reduced ALT, GGT, urea, and creatinine levels towards normalcy in diabetic rats. 

### 3.7. Gene Expression Study Using GeXP Analyzer

Current study was conducted to determine the mechanism of *C. latifolia* fruit:root extract in alleviating insulin resistance through altering expression of genes involved in glucose and lipid metabolisms. In muscle tissue, the expression of *IRS1, IGF, GLUT4, PPAR*α*, PPAR*γ*, AdipoR1, AdipoR2*, *leptin, LPL,* and *lipase *genes in obese and diabetic control rats was significantly (*P* < 0.05) lower than that in normal rats either in muscle or adipose tissues. However, *C. latifolia* fruit:root extract significantly (*P* < 0.05) increased the mRNA expression of these genes in diabetic rats as compared to diabetic control rats in Group 3 (Tables 7 and 8). 

## 4. Discussion

In order to find the similarity in the development of diabetes in humans, induction of HFD with low dose STZ is the preferred method. However, the rats with HFD will increase their energy expenditure and then lead towards the progression of insulin resistant in the organs. Moreover, low dose STZ induced hyperglycemia in rats where it defects the secretion of insulin [5]. This progressive development of type 2 DM is similar to humans where several researchers agree [36]. In this study, high fat diet was formulated according to Levin et al. (1989) and this diet contains 34% of fat out of 100 g of total HFD diet [34]. There was 88% increase in fat compared to normal diet. Study done by Warwick et al. (2002) has reported that the amount of fat needed in high fat diet must be in the range 30% to 60% out of total diet and that is because this amount allows the changes of body weight composition, endocrine secretion and metabolic [37]. Thus, this indicates that, the findings of our HFD are in agreement's with Warwick et al. findings. The current result clearly demonstrated that body weight of obese rats (Group 2) increased over normal rats and this is due to high fat intake. Meanwhile, body weights among diabetic control rats were lower compared to obese rats due to STZ injection. Chatterjee and Shinde (2002) mentioned in their report that STZ causes reduction in body weight due to the loss of tissue protein and increased muscle wasting [38–40]. However, *C. latifolia* fruit:root extract caused weight loss in diabetic rats in Groups 4, 5, and 6 compared to diabetic control rats. This could be due to lipid lowering activity by *C. latifolia* fruit:root extract [41]. Besides, body weight among diabetic rats treated with glibenclamide increased compared to diabetic control rats and this indicates that *C. latifolia* fruit:root extract is better than glibenclamide in order to prevent body weight gain in diabetic models. 

Diabetic rats induced by high fat diet with combination of low dose STZ have closely mimicked the natural process of the diabetic occurrence and metabolic disturbance in human diagnosis as type 2 diabetes [42]. In the present study, diabetic model that has been developed using high fat diet with combination of 40 mg of STZ also shows the same symptoms as reported by Unger et al. (2010) [13]. Blood glucose level in diabetic rats was increased and this finding supported the findings of Poitout and Robertson (2002), where they have mentioned that STZ causes destruction of pancreatic *β*-cells and it makes the cells less active to be sensitive enough towards insulin for glucose uptake and this will cause high glucose concentration in blood [43]. Besides, Bansal et al. (2012) Insulin-mediated glucose uptake mentioned that HFD fed with STZ combination causes hyperglycemia in rats [44]. However, aqueous extract of the *C. latifolia* fruit:root exhibited a hypoglycemic effect and significantly (*P* < 0.05) decreased the glucose level. Extract at 200 mg/kg bw has showed a higher decrease compared to other concentrations. It has decreased 64.5% inblood glucose level and followed by 100 mg/kg bw and 50 mg/kg bw. The ability of *C. latifolia* fruit:root extract in reducing glucose level was in agreement with previous finding in an *in vitro* study (patent pending). *C. latifolia* increased glucose uptake activity in adipocyte and myotube cells at basal and through insulin-mediated glucose/STZ is being uptake : glucose/STZ is being translocation. Besides, *C. latifolia* also possesses sensitize and insulin mimicking actions in order to stimulate glucose uptake activity. In insulin presence, *C. latifolia* extracts might sensitizing insulin signaling cascade and stimulates translocation of glucose transporter GLUT4 into plasma membrane and then glucose is being uptake into adipose and muscle through phosphatidylinositol 3-kinase (PI3K) pathway [45].

The abnormality in lipid metabolism in type 2 diabetes mellitus has caused hyperlipidemia in diabetic patient. Thus, diabetic rats that have been induced by HFD with combination of low dose STZ also showed similar situation, hyperlipidemia. This finding further support the idea of Lombardo and Chicco (2006) where it is shown that those rats administrated with HFD cause dyslipidemia and other syndromes in diabetices [46]. Besides, defect in insulin secretion due to STZ also causes defect in lipogenic activity. Insulin plays an important role in stimulating lipogenesis in mammals, by low secretions of insulin it implicates of high level of lipid in plasma [47–49]. Furthermore, plasma colour has turned to opaque due to lipid presence. However, after 4 weeks of treatment with 200 mg/kg bw of *C. latifolia* fruit:root extracts, TC, TG, and LDL levels were significantly (*P* < 0.05) decreased and HDL level was increased compared to pretreatment levels. In spite of that, opaque colour in plasma was turned into clear in plasma sample in Groups 4, 5, and 6.

Disruption of pancreatic *β*-cells by STZ in diabetic rats has caused insufficient insulin secretion in blood [42]. Thus, this STZ is being uptake into pancreatic cells by GLUT2 and it causes DNA damage via reactive oxygen species generation [42]. However, *C. latifolia* fruit:root extracts in all concentration have prevented further disruption of cells. It indicates that antioxidant properties of *C. latifolia* have scavenged free radicals which cause oxidative stress in cells. Moreover, adiponectin secretion was also decreased in diabetic control rats. This finding is in agreement with Yang et al.'s (2006) finding which showed that rats treated with HFD for 4 weeks showed significantly decreased in adiponectin [50]. However, after 4 weeks of treatment with *C. latifolia* fruit:root extracts, adiponectin levels were increased. The possible mechanism of *C. latifolia* towards this increasing is due to improvement of insulin secretion [41]. Besides, *C. latifolia* extracts also possess insulin mimicking properties which trigger the adiponectin secretion in adipose tissues. These results suggest that amelioration of insulin and adiponectin secretions by *C. latifolia* fruit:root extract may be a key to decrease glucose and lipid levels in diabetic rats. 

Hepatotoxicity and nephropathy are complications from T2DM. Hepatocytes damaged due to hepatotoxicity cause ALT and GGT enzymes leaking out into the blood circulation. Present findings showed that those enzymes were significantly higher in diabetic rats in Groups 2 and 3. These findings are consistent with those of Bolkent et al. (2004) who found that high cholesterol level could cause damage to the liver [51]. Since in our study diabetic rats also showed high cholesterol level, so it supports the idea that high cholesterol level leads to liver damage. Meanwhile, result also shown that creatinine and urea levels were significantly high (*P* < 0.05) in diabetic rats. This finding is similar to that of Sugano et al. (2006) where they had developed nephropathy in T2DM rats model using the same method as in our study and it indicates that HFD and low dose STZ can produce naturally nephropathy symptom similar in humans [52]. However, ALT, GGT, creatinine, and urea levels in diabetic rats treated with *C. latifolia* fruit:root extract were lower than diabetic control rats. This finding showed that no lethality or toxicity was observed during 4 weeks of intervention with *C. latifolia* fruit:root extracts on diabetic rats. Besides, it indicates that *C. latifolia* fruit:root extract prevents further defect in kidney and liver functions.

High fat diet influences human health status by changing cellular function during transcription process [53]. According to Rakhshandehroo et al. (2010), high fat diet may downregulate several transcription factors such as nuclear receptor (PPAR) and sterol regulatory binding proteins (GLUT) [53]. IRS-1, GLUT4, and IGF-1 are responsible of glucose metabolisms. Binding of insulin on IRS initiates PI3K substrates and triggers the activation of glucose transporter (GLUT4) vesicles to plasma membrane for glucose uptake [54]. Meanwhile, IGF-1 also has direct effect in order to trigger insulin sensitivity and regulate glucose uptake similar to insulin. Although insulin and IGF has similar mechanism in inducing glucose uptake, several studies showed that IGF is more efficient than insulin [55]. Thus, our finding showed that *IRS-1, IGF-1,* and *GLUT4* genes have been downregulated in obese and diabetic control rats. This finding was associated with the occurrence of defect insulin secretion and insulin resistance in obese and diabetic control rats [56]. However, *C. latifolia* fruit:root extract increased *IRS-1, IGF-1,* and *GLUT4* genes expression in diabetic rats. The augmented expression of these genes is due to the ability of *C. latifolia* fruit:root extract to increase insulin secretion and sufficient insulin will trigger the expression of *IRS-1* gene. Moreover, expression of *IGF-1* gene was higher compared to IRS-1. This indicates that *C. latifolia* fruit:root extract is more potent to improve glucose metabolism through IGF-1 action than IRS-1. Besides, it also indicates that *C. latifolia* fruit :  root extract could act in two different situations, early and late phase of type 2 diabetes where pancreatic cells could not keep up with demand. However, further study needs to be done to study the mechanism involved. 

Meanwhile, *PPAR*γ** and *PPAR*α** were also downregulated in obese and diabetic control rats. Research done by Petersson et al. (2009) has also found that the occurrence of insulin resistance, hyperglycemia, and dyslipidemia in diabetes subjects is because of downregulation of several genes such as *IGF-1, PPAR,* and *GLUT* families [57, 58]. Four weeks of intervention with *C. latifolia* fruit:root extract caused upregulation of *PPAR*γ**gene. According to Pita et al. (2012), the upregulation of *PPAR*γ** will stimulate *IRS-1, IGF-1, GLUT4, AdipoR1, AdipoR2, LPL, PPAR*α*, leptin,* and *lipase* genes expression in the treated diabetic rats [59] and their findings support our results where the same genes are also being upregulated. Besides, *C. latifolia* fruit:root extract might have similar action to thiazolidinedione (TZD) which possess PPAR**γ** ligand-binding activity and stimulate PPAR**γ** transcription [60]. 

On the other hand, *AdipoR1, AdipoR2, leptin, LPL,* and *lipase* genes were also downregulated in diabetic control rats either in muscle and adipose tissues. According to Kadowaki and Yamauchi (2005), AdipoR1 and AdipoR2 ameliorate defect in glucose and lipid metabolism through different pathways [61]. AdipoR1 activates the AMPK pathway in order to reduce hepatic glucose production and increase FA oxidation. Meanwhile, AdipoR2 activates PPAR*α* pathway [61]. In the present study, *C. latifolia* fruit:root extract increased regulation of *AdipoR1* and *AdipoR2* genes. These genes might be upregulated during adipocyte differentiation or through other genes expression. According to Pita et al. (2012), the enhancement of adiponectin receptors expression is correlated with increasing AMPK activity which is necessary for adiponectin action [59]. Our finding is parallel to their finding where *C. latifolia* fruit:root extract augmented glucose metabolism genes such as *IRS-1, IGF-1,* and *GLUT4* and it might increase AMPK activity in diabetic rats and lead to upregulation of *AdipoR1* and *AdipoR2* genes. 

Several studies have indicated the importance of PPAR*α* in lipid and glucose metabolisms [62]. According to Gross and Staels (2007), activation of PPAR*α* may express other genes involved in lipid and lipoprotein metabolism [62]. Besides, PPAR*α* is also responsible of glucose homeostasis and it directly regulates gluconeogenesis due to pyruvate dehydrogenase kinase 4 expression [63]. Apart from that, PPAR*α* has the ability to prevent insulin resistance in diabetic due to the increase of fatty acids oxidation in pancreas, muscle and liver [64]. In spite of that, it also improves insulin secretion by preventing lipotoxicity and glucotoxicity in pancreatic cells [65]. In the present study, *C. latifolia* fruit:root extracts upregulate PPAR*α* transcription in both muscle and adipose tissues of diabetic rats. The activation of PPAR*α* will lead to high level of HDL and low level of TG in diabetic model [53]. This finding is in agreement with the present data where TG level was decreased and HDL level was increased in treated diabetic rats after 4 weeks of intervention with *C. latifolia* fruit:root extracts. Moreover, the upregulation of PPAR*α* will initiate the regulation of genes involved in lipoprotein metabolism such as LPL [52]. Thus, it compliments with the recent study where *C. latifolia* fruit:root extracts increased LPL expression in diabetic rats. The possible mechanism that can be drawn from this finding is that *C. latifolia* fruit:root extract indirectly increased LPL expression via activation of PPAR*α* through adipocytokine signaling pathway. Apart from that, LPL expression might be another possible explanation on how opaque colour in plasma turned into clear. Lipid content in plasma will be hydrolyzed by LPL and converted into TG for storage [66]. Besides, slight increase in body weight among treated diabetic rats was due to upregulation of leptin expression.

## 5. Conclusion

The present study demonstrates that *C. latifolia* fruit:root extract reduced glucose and lipid levels in diabetic rats. Meanwhile, insulin and adiponectinlevels were increased. The extract exerts antidiabetic and hypolipidemic effects by stimulating PPAR*γ*, IRS-1, IGF-1, GLUT4, AdipoR1, AdipoR2, LPL, PPAR*α*, leptin, and lipase expressions in adipose and muscle tissues. The antidiabetic and hypolipidemic activities of *C. latifolia* fruit:root support its potential as a therapeutic option for diabetes and its complication. 

## Figures and Tables

**Table 1:** Nutrient composition of NPD and HFD.

Nutrient	% in 100 g of NPD	% in 100 g of HFD
Fat	4	34
Fiber	5	3
Protein	14	16
Carbohydrate	72	42
Mineral and vitamin mix	5	5

Total calories (kcal/100 g)	380	538

**Table 2:** Sequence of primers used.

Accession number	Left sequence w/universals	Right sequence w/universals
NM_012969 (IRS1)	AGGTGACACTATAGAATAACCCT AGACCCACTGCCTTT	GTACGACTCACTATAGGGATGGAGGAAGCA AGCAGAAAT
NM_013124 (PPAR*γ*)	AGGTGACACTATAGAATAGATCCTCCTG TTGACCCAGA	GTACGACTCACTATAGGGATCAAAGGAATGGG AGTGGTC
NM_178866 (IGF)	AGGTGACACTATAGAATACCGCTGAAGCC TACAAAGTC	GTACGACTCACTATAGGGAGCTCAAGCAGCAA AGGATCT
NM_012751 (GLUT4)	AGGTGACACTATAGAATAAATGACTGAGGGG CAAAATG	GTACGACTCACTATAGGGAGGGTAAGAGGAA GGCAGGAC
NM_207587 (AdipoR1)	AGGTGACACTATAGAATAGGACTTGGCTTG AGTGGTGT	GTACGACTCACTATAGGGACGGAATTCCTGTA GGTTGGA
Kan^r^	AGGTGACACTATAGAATAATCATCAGCA TTGCATTCGATTCCTGTTTG	GTACGACTCACTATAGGGAATTCCGACTCGT CCAACATC
NM_013196 (PPAR*α*)	AGGTGACACTATAGAATACTCGTGCAGGTCA TCAAGAA	GTACGACTCACTATAGGGAGCCTCTGATCACC ACCATTT
NM_013076 (Leptin)	AGGTGACACTATAGAATACAAAACGTGC TGCAGATAGC	GTACGACTCACTATAGGGACATTCAGGGCTA AGGTCCAA
NM_012598 (LPL)	AGGTGACACTATAGAATAACTCGCTCTCA GATGCCCTA	GTACGACTCACTATAGGGACTGACCAGCGGA AGTAGGAG
NM_012859 (Lipase)	AGGTGACACTATAGAATACCTTCGGGGAA CACTACAAA	GTACGACTCACTATAGGGACCAAGGGAGGTG AGATGGTA
NM_023964 (GAPDH)	AGGTGACACTATAGAATAATCAATGGATTT GGACGCAT	GTACGACTCACTATAGGGAAGCTCCAGGGGA TTTCCTTA
BC168964	AGGTGACACTATAGAATACGGAAGAAGGCT CTTGAAAA	GTACGACTCACTATAGGGACGCCACCCTCTT CATCTCTA
NM_001037979 (AdipoR2)	AGGTGACACTATAGAATACGGTGTACTGCCA CTCAGAA	GTACGACTCACTATAGGGAGCAAGGTAGGGAT GATTCCA

**Table 3:** Energy contributed from NPD and HFD.

Nutrient	% in 100 g of NPD	% energy in NPD	% in 100 g of HFD	% energy in HFD
Fat	4	9.5	34	56.9
Protein	14	14.7	16	11.9
Carbohydrate	72	75.8	42	31.2

Percentage (%) of energy was measured by kilocalories.

**Table 4:** The plasma glucose level and percentage of plasma glucose changes.

Plasma glucose level (mmol/L)	Rat group						
and percentage of glucose level (%)	Group 1	Group 2	Group 3	Group 4	Group 5	Group 6	Group 7
Plasma glucose level at week 0 (mmol/L)	5.21 ± 2.18	9.52 ± 4.31	21.61 ± 5.56	20.41 ± 5.81	19.75 ± 5.18	21.55 ± 4.64	22.26 ± 6.12
Plasma glucose level at week 4 (mmol/L)	5.34 ± 3.57	11.69 ± 5.31	25.47 ± 4.38*	13.5 ± 21.13**	12.70 ± 15.72**	13.10 ± 19.27**	16.8 ± 17.22**
Plasma glucose changes from week 0 to week 4	2.4	18.6	15.2	−51.6	−54.3	−64.5	−32.4

Data are means ± SD for plasma glucose level of normal rats (Group 1), obese rats (Group 2), diabetic control rats (Group 3), diabetic rats treated with 50 mg/kg bw of *C. latifolia* fruit:root extracts (Group 4), diabetic rats treated with 100 mg/kg bw of *C. latifolia* fruit:root extracts (Group 5), diabetic rats treated with 200 mg/kg bw of *C. latifolia* fruit:root extracts (Group 6), and diabetic rats treated with 10 mg/kg bw of glibenclamide (Group 7) at weeks 0 and 4.

*Are significantly different at *P* < 0.05 compared with diabetic control, that is, Group 3.

**Are significantly different at *P* < 0.001 compared with diabetic control, that is, Group 3.

− Indicates the reduction in plasma glucose.

**Table 5:** Lipid profiles of rats group.

Group	Total cholesterol (mmol/L)		TG (mmol/L)		LDL (mmol/L)		HDL (mmol/L)	
	Before	After	Before	After	Before	After	Before	After
1	1.73 ± 0.29	1.62 ± 0.18	0.48 ± 0.26	0.57 ± 0.11	0.54 ± 0.31	0.57 ± 0.42	0.57 ± 0.22	0.53 ± 0.24
2	2.65 ± 0.11	3.38 ± 0.16*	0.43 ± 0.13	1.17 ± 0.36*	0.77 ± 0.11	0.92 ± 0.26	0.54 ± 0.36	0.26 ± 0.09
3	2.17 ± 0.12	2.53 ± 0.24	0.60 ± 0.16	0.87 ± 0.20	0.70 ± 0.06	1.00 ± 0.10*	0.52 ± 0.05	0.26 ± 0.15
4	3.43 ± 0.15*	1.49 ± 0.22*	0.53 ± 0.09	0.64 ± 0.30	1.34 ± 0.42*	0.85 ± 0.06	0.40 ± 0.15	0.75 ± 0.21*
5	2.20 ± 0.23	1.21 ± 0.16*	0.52 ± 0.03	0.67 ± 0.13*	0.66 ± 0.16	0.59 ± 0.38*	0.37 ± 0.29*	0.82 ± 0.19*
6	3.24 ± 0.10*	1.17 ± 0.28*	0.60 ± 0.24	0.69 ± 0.19*	1.07 ± 0.15*	0.56 ± 0.13*	0.34 ± 0.15*	0.69 ± 0.20*
7	2.96 ± 0.14	1.35 ± 0.16*	0.45 ± 0.24	0.59 ± 0.13*	0.68 ± 0.17	0.76 ± 0.24	0.38 ± 0.11*	0.77 ± 0.27*

Data are means ± SD for lipid profiles of normal rats (Group 1), obese rats (Group 2), diabetic control rats (Group 3), diabetic rats treated with 50 mg/kg bw of *C. latifolia* fruit:root extracts (Group 4), diabetic rats treated with 100 mg/kg bw of *C. latifolia* fruit:root extracts (Group 5), diabetic rats treated with 200 mg/kg bw of *C. latifolia* fruit:root extracts (Group 6), and diabetic rats treated with 10 mg/kg bw of glibenclamide (Group 7) at weeks 0 (before) and 4 (after).

*Are significantly different at *P* < 0.05 compared with diabetic control, that is, Group 3.

**Table 6:** Effect of *C. latifolia* extracts on ALT, GGT, urea, and creatinine levels in HFD and low dose STZ induced diabetic rats.

Group	ALT (U/L)		GGT (U/L)		Urea (mmol/L)		Creatinine (mmol/L)	
	Before	After	Before	After	Before	After	Before	After
1	75.12 ± 2.34	109.24 ± 3.42	6.73 ± 1.19	5.04 ± 0.69	5.51 ± 0.33	5.89 ± 0.51	31.30 ± 1.11	30.97 ± 2.52
2	88.24 ± 1.55	114.81 ± 5.10	8.65 ± 2.12	10.32 ± 0.52*	5.07 ± 0.14	5.82 ± 0.37	49.40 ± 2.54	62.90 ± 3.46*
3	89.12 ± 3.42	149.65 ± 4.26*	13.11 ± 1.31*	27.88 ± 2.37*	4.53 ± 0.35	6.83 ± 0.40	52.85 ± 2.68	72.10 ± 2.58*
4	77.50 ± 2.27	76.49 ± 1.33*	9.53 ± 1.34	5.39 ± 0.89*	9.61 ± 0.16*	6.66 ± 0.25	58.80 ± 2.34	47.80 ± 1.19
5	76.21 ± 3.17	84.77 ± 2.50*	9.07 ± 1.28	3.18 ± 1.41	12.55 ± 0.42	5.66 ± 0.26*	59.13 ± 1.19	51.40 ± 0.77
6	80.50 ± 1.24	78.10 ± 2.46*	9.25 ± 1.31	5.10 ± 0.17*	9.15 ± 0.36	6.65 ± 0.41	51.93 ± 0.76	42.60 ± 0.15
7	85.74 ± 5.31	83.10 ± 3.26*	8.90 ± 1.19	5.84 ± 0.21*	14.62 ± 0.54*	4.98 ± 0.33	57.63 ± 0.50	56.21 ± 0.32

Data are means ± SD for ALT, GGT, urea and creatinine levels of normal rats (Group 1), obese rats (Group 2), diabetic control rats (Group 3), diabetic rats treated with 50 mg/kg bw of *C. latifolia* fruit:root extracts (Group 4), diabetic rats treated with 100 mg/kg bw of *C. latifolia* fruit:root extracts (Group 5), diabetic rats treated with 200 mg/kg bw of *C. latifolia* fruit:root extracts (Group 6), and diabetic rats treated with 10 mg/kg bw of glibenclamide (Group 7).

Significant difference is at *P < 0.05 when compared with diabetic control rats (Group 3).

**Table 7:** Expression of candidate genes in muscle tissue.

Group	IRS1	IGF	GLUT4	PPAR*γ*	PPAR*α*	AdipoR1	AdipoR2	Leptin	LPL	Lipase	Gapdh
Group 1	1.8260	1.9954	0.9704	1.5499	1.3345	1.4130	1.2861	1.3915	0.5263	0.5804	0.7407
Group 2	0.8634	0.9465	0.5567	0.4557	0.7075	0.5208	0.4300	2.5701*	0.9524	0.9544	0.9787
Group 3	0.5530	0.6140*	0.5163	0.3232	0.5192	0.4040	0.3334	0.6173*	0.7185	0.7652	1.0191
Group 4	1.3284*	5.2166*	1.3854*	2.0186*	3.0955*	1.4058*	1.5691*	2.1324*	0.8530	1.9529*	1.1178
Group 5	1.4668*	5.3301*	1.9030*	2.4303*	3.2608*	1.4815*	1.5556*	2.2548*	0.7183	1.6386*	1.2593*
Group 6	1.7949*	5.6444*	2.8137*	3.5724*	3.8732*	2.5713*	2.8885*	3.2222*	0.5769	2.0382*	1.3244*
Group 7	1.2346*	4.0180*	1.0819*	0.3098	0.2155	0.8305	1.3058*	0.3030	1.1089*	0.9428	1.2346*

Data are means ± SD for expression of candidate genes in muscle tissue of normal rats (Group 1), obese rats (Group 2), diabetic control rats (Group 3), diabetic rats treated with 50 mg/kg bw of *C. latifolia* fruit:root extracts (Group 4), diabetic rats treated with 100 mg/kg bw of *C. latifolia* fruit:root extracts (Group 5), diabetic rats treated with 200 mg/kg bw of *C. latifolia* fruit:root extracts (Group 6), and diabetic rats treated with 10 mg/kg bw of glibenclamide (Group 7).

Significant difference is at *P < 0.05 when compared with diabetic control rats (Group 3).

**Table 8:** Expression of candidate genes in adipose tissue.

Group	IRS1	IGF	GLUT4	PPAR*γ*	PPAR*α*	AdipoR1	AdipoR2	Leptin	LPL	Lipase	Gapdh
Group 1	1.5371	1.8971	1.4174	1.5781	1.7697	1.6875	1.2268	0.9548	0.5681	0.6158	0.9851
Group 2	0.4879	0.7495	0.5275	0.2414	0.4568	0.5967	0.5692	3.7523*	0.8751	0.7681	0.8562
Group 3	0.4378	0.5644	0.3194	0.2089	0.3699	0.4298	0.4692	0.5689	0.7521	0.5821	1.5240
Group 4	1.3280*	1.7462*	2.0911*	2.9885*	1.5538*	2.8792*	2.1732*	1.5982*	3.5418*	1.5556*	1.4338
Group 5	1.7888*	2.3779*	1.4028*	1.3769*	0.8992*	2.0781*	2.3976*	2.8826*	2.4351*	1.9261*	1.3561
Group 6	1.6971*	2.5811*	1.0247*	1.5571*	1.6982*	2.1169*	3.4691*	2.9871*	2.9984*	2.4861*	1.5983
Group 7	1.1555*	0.7924	1.3333*	1.8634*	0.6282*	1.7295*	1.8059*	1.055	0.8564	0.7539	1.2374

Data are means ± SD for expression of candidate genes in adipose tissue of normal rats (Group 1), obese rats (Group 2), diabetic control rats (Group 3), diabetic rats treated with 50 mg/kg bw of *C. latifolia* fruit:root extracts (Group 4), diabetic rats treated with 100 mg/kg bw of *C. latifolia* fruit:root extracts (Group 5), diabetic rats treated with 200 mg/kg bw of *C. latifolia* fruit:root extracts (Group 6), and diabetic rats treated with 10 mg/kg bw of glibenclamide (Group 7).

*Indicates *P* < 0.05 compared with diabetic control rats (Group 3).
